# Mechanical effects of midwall fibrosis in non-ischemic dilated cardiomyopathy

**DOI:** 10.1186/1532-429X-16-S1-P308

**Published:** 2014-01-16

**Authors:** Robin J Taylor, Fraz Umar, Lai Sze Lin, Amar Ahmed, William E Moody, Berthold Stegemann, John N Townend, Richard P Steeds, Francisco Leyva

**Affiliations:** 1 Centre for Cardiovascular Sciences, University of Birmingham, Birmingham, UK; 2 Department of Cardiology, Queen Elizabeth Hospital, Birmingham, UK; 3 Bakken Research Center, Medtronic Inc, Maastricht, Netherlands

## Background

In patients with non-ischemic dilated cardiomyopathy (NIDCM), mid-wall fibrosis (MWF) is associated with a higher risk of hospitalizations and death from pump failure and sudden cardiac death. The mechanical effects of MWF remain unexplored. Strain measures derived from feature tracking-CMR (FT-CMR) have been validated against myocardial tagging.

## Methods

Patients (n = 84, age: 57.7 ± 14.7 yrs, [mean ± SD], LVEF: 25.7 ± 11.1%) with newly diagnosed NIDCM underwent late gadolinium enhancement CMR (inversion-recovery technique 10 min after the administration of gadolinium-DTPA (0.1 mmol/kg). Peak systolic circumferential (Ε_cc_) and radial (Ε_rr_) strain were assessed using FT-CMR of the mid-cavity LV short-axis cine and peak systolic longitudinal strain (Ε_ll_) was assessed from the horizontal long axis cine.

## Results

**Figure 1:**
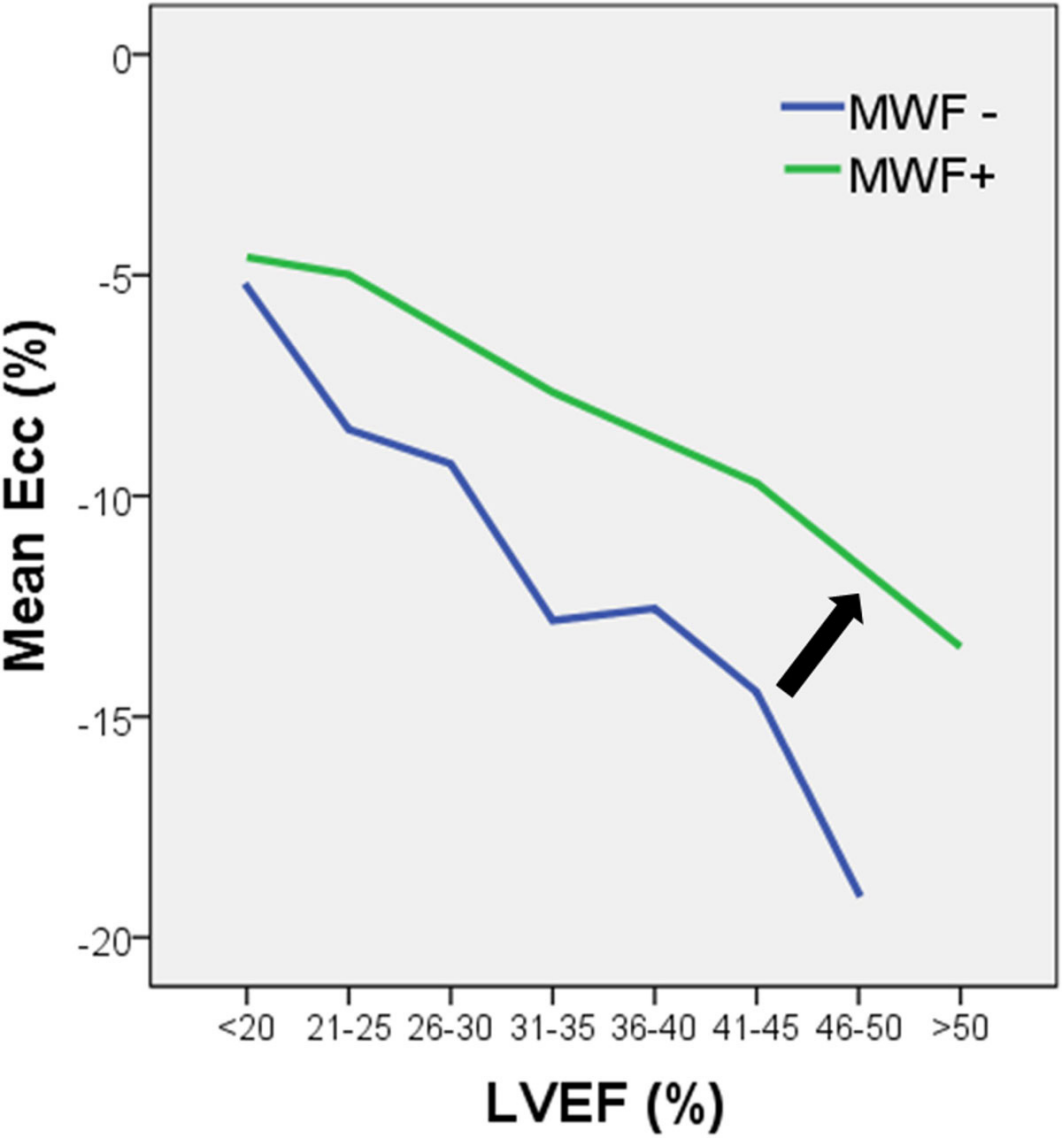
**The relationship between Εcc and LVEF**.

Patients with MWF (n = 21) had a similar LVEF to patients without (n = 63) (22.1 ± 11.7 vs 26.9 ± 10.9%, p = 0.85). Patients with MWF had reduced Ε_cc _(-5.9 ± 2 vs -9.4 ± 4.9%, p = 0.001), but similar Ε_rr _(12.5 ± 7.9% vs. 15.9 ± 9.4%, p = 0.31) and Ε_ll _(-7.7 ± 3.5% vs -9.0 ± 6.0%, p = 0.18). In patients with similar LVEF, Ε_cc _was consistently lower in those with MWF compared to those without, across a broad range of myocardial function. As shown below, MWF has the effect of altering the relationship between Ε_cc _and LVEF (see Figure 1).

## Conclusions

In patients with NICM and comparable LVEF, MWF is associated with impaired myocardial contraction in a circumferential direction. These findings are likely to be useful in the prognostic stratification of patients with NICM. They may also account for the recently recognised negative effects of MWF in patients undergoing CRT.

## Funding

None.

